# Preparation of Chito-Oligomers by Hydrolysis of Chitosan in the Presence of Zeolite as Adsorbent

**DOI:** 10.3390/md14080043

**Published:** 2016-07-23

**Authors:** Khalid A. Ibrahim, Bassam I. El-Eswed, Khaleel A. Abu-Sbeih, Tawfeeq A. Arafat, Mahmoud M. H. Al Omari, Fouad H. Darras, Adnan A. Badwan

**Affiliations:** 1 Department of Chemical Engineering, Faculty of Engineering, Al-Hussein Bin Talal University, P.O. Box 20, Ma`an 71111, Jordan; khalida@ahu.edu.jo; 2 College of Engineering, King Saud University, P.O. Box 800, Riyadh 11421, Saudi Arabia; 3 Department of Basic Sciences, Zarqa College, Al-Balqa Applied University, P.O. Box 313, Zarqa 13110, Jordan; bassameswed@bau.edu.jo; 4 Department of Chemistry, Faculty of Science, Al-Hussein Bin Talal University, P.O. Box 20, Ma`an 71111, Jordan; abusbeih@ahu.edu.jo; 5 Department of Pharmaceutical Medicinal Chemistry and Pharmacognosy, Faculty of Pharmacy and Medical Technology, Petra University, P.O. Box 961343, Amman 11196, Jordan; tarafat@uop.edu.jo; 6 Research and Innovation Center (RIC), The Jordanian Pharmaceutical Manufacturing Co., P.O. Box 94, Naor 11710, Jordan; momari@jpm.com.jo (M.M.H.A.O.); fdarras@jpm.com.jo (F.H.D.); 7 The Jordanian Pharmaceutical Manufacturing Co., P.O. Box 94, Naor 11710, Jordan

**Keywords:** chitosan, chito-oligomers, zeolite, depolymerization, hydrolysis

## Abstract

An increasing interest has recently been shown to use chitin/chitosan oligomers (chito-oligomers) in medicine and food fields because they are not only water-soluble, nontoxic, and biocompatible materials, but they also exhibit numerous biological properties, including antibacterial, antifungal, and antitumor activities, as well as immuno-enhancing effects on animals. Conventional depolymerization methods of chitosan to chito-oligomers are either chemical by acid-hydrolysis under harsh conditions or by enzymatic degradation. In this work, hydrolysis of chitosan to chito-oligomers has been achieved by applying adsorption-separation technique using diluted HCl in the presence of different types of zeolite as adsorbents. The chito-oligomers were retrieved from adsorbents and characterized by differential scanning calorimetry (DSC), liquid chromatography/mass spectroscopy (LC/MS), and ninhydrin test.

## 1. Introduction

**Figure 1:**
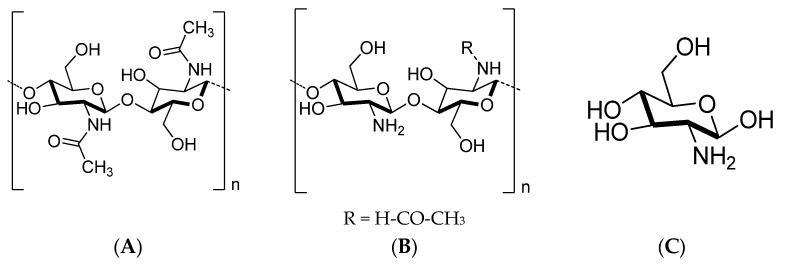
Chemical structure of (**A**) chitin; (**B**) chitosan; and (**C**) glucosamine.

Chitosan—among various renewable polymers—is one of the most commercially important biocompatible polymers from an environmental or biomedical point of view [1,2,3]. It is a linear copolymer of (1→4)-linked 2-acetamido-2-deoxy-β-d-glucan (GlcNAc) and 2-amino-2-deoxy-β-d-glucan (GlcN) units in varying proportions (Figure 1) [4,5]. Naturally, chitosan is produced by the hydration of a nitrogenous polysaccharide chitin (Figure 1), which is considered as the main building component of crustacean shells [6,7].

Recently, various N-containing products have been prepared from chitin [8,9,10,11,12]. For example, chitin has been converted to 3-acetamido-5-acetylfuran by using *N*-methyl-2-pyrrolidone [8] and ionic liquids [9] as solvents. Additionally, it has been converted to its corresponding amide/amino substituted sugar alcohols, smaller C_2_–C_4_ polyols and *N*-acetylmonoethanolamine over transition metal catalysts and hydrogen in water [10]. Two major products, namely hydroxyethyl-2-amino-2-deoxyhexopyranoside and hydroxyethyl-2-acetamido-2-deoxyhexopyranoside, have been obtained by acid-catalyzed liquefaction of chitin in ethylene glycol [11]. Furthermore, N-containing carbon materials have been prepared by carbonization of chitin, which used as adsorbents to remove toxic heavy metals and in styrene epoxidation [12]. These findings offer opportunities to convert biomass such as chitin into value-added, renewable N-containing materials [13].

The cationic nature of chitosan made it a unique polysaccharide, which is distinguished among other polysaccharides [14]. It is mainly obtained at the synthetic scale by deacetylation of chitin, and the process of deacetylation is carried out to different degrees depending upon the targeted applications, so numerous products with different degrees of deacetylation (DD) can be obtained. The physiological properties of chitosan, especially solubility, are determined by its molecular weight and degree of deacetylation. Chitosan is a water insoluble polysaccharide while, because of its cationic nature, is soluble in dilute acidic solutions [15]. The insoluble nature of chitosan in neutral pH restricted its use in solution for physiological applications in the medical and food industries [16].

Chito-oligomers are the hydrolysates of chitosan, mainly made up of -1,4 linked d-glucosamine and partially of -1,4 linked *N*-acetyl-d-glucosamine. Previously, many studies showed that chito-oligomers have significant potential in medicine and food fields due to their wide bioactivity, such as antibacterial, antifungal, antitumor activity, radical scavenging, antimicrobial activity, immunity modulatory effect, and wound healing [15,17,18,19]. Glucosamine is a monomer of chito-oligomers (Figure 1) which has a growing market due to its use for the treatment of osteoarthritis [20]. However, the in-depth knowledge of the mode of action of chito-oligomers is still limited because their biological activity has often been determined using heterogeneous and/or relatively poorly characterized oligomer mixtures [15].

Different methods have been described in the literature to prepare chito-oligomers from chitosan by enzymatic and chemical methods [15,16,19,21,22]. Enzymatic methods are selective and simple but their commercial applications are limited due to the cost, low yield, and limited availability of chitosan-specific enzymes [21,22]. The chemical methods include depolymerization of chitosan by a hydrolysis reaction, mainly using concentrated HCl [23], nitrous acid [24], fluorolysis in anhydrous hydrogen fluoride [25], and oxidative-reductive reaction by hydrogen peroxide [26]. Additionally, a few total chemical syntheses of chito-oligomers involving multiple protection and deprotection steps have also been reported [27,28]. Preparation of chito-oligomer mixtures from chitosan by physical methods (hydrothermal, microwave, ultrasonication, and gamma-ray) was reviewed by Yin *et al.* [29]. This subject was investigated in a limited number of studies and still uncovered [30].

Zeolites are well-known as valuable crystalline solids with framework structures containing discrete micropores of molecular dimensions that accommodate exchangeable extra-framework cation sites [31,32]. In terms of host-guest interactions, zeolites can be viewed as host frameworks with structurally intact and immutable three-dimensional (3D) structures [33,34]. Zeolites are widely used in commercial applications as petroleum refining, petrochemical industry, and fine chemical industry, as sorbents for small-molecule separation processes and as ion-exchangers in detergents [35,36,37]. Due to all of the properties mentioned above, zeolites are employed as adsorbent materials in this work to shift the equilibrium of the hydrolysis reaction toward the formation of chito-oligomers in an adsorption-separation technique.

Concentrated HCl hydrolysis of chitosan to chito-oligomers is the best known and applied chemical method [16,19,22,23,38]. Table 1 summarizes the reaction conditions, reagents, degree of acetylation (DA) of the chitosan used, degree of polymerization (DP), and characterization methods of the produced chito-oligomers (see Table 1). All reported methods started from almost deacetylated chitosan hydrolysis; the general protocol employed concentrated HCl for hydrolysis of chitosan, which has several disadvantages like harsh conditions, many purification steps to remove the strong acid and low yields of chito-oligomers obtained from such reaction conditions.

Due to all of the mentioned disadvantages of using classical hydrolysis procedures of chitosan to its chito-oligomers, this work presents a novel application of adsorption reaction techniques to hydrolyze chitosan to chito-oligomers using diluted HCl in the presence of zeolite as adsorbent. The chito-oligomers obtained from the protocol used in the present work were subjected to a number of identification and characterization tests to understand the nature of the chito-oligomers obtained and to evaluate the hydrolysis technique applied in this work against the conventional hydrolysis methods used before.

## 2. Results and Discussion

### 2.1. Hydrolysis of Chitosan to Its Chito-Oligomers

**Scheme 1:**
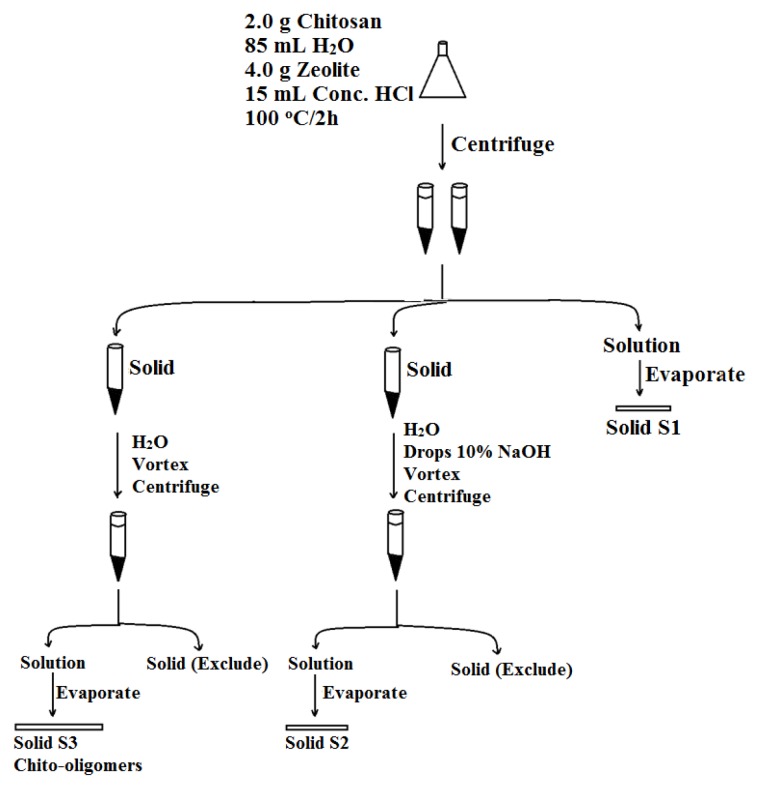
Preparation protocol of chito-oligomers.

As a result of the designed separation technique, starting from 2.0 g chitosan and 4.0 g of zeolite (different types: 10Ǻ, 5Ǻ, 3Ǻ, Table 2), in 100 mL 1.8 M HCl (85 mL H_2_O and 15 mL conc. HCl), the reaction mixture was stirred in a water bath under reflux for two hours, three solid samples were then collected and named accordingly S1, S2, and S3 as described briefly in Scheme 1.

Three different tests (Table 3) were performed as preliminary tests to determine the chito-oligomers content of the hydrolysis reactions products S1, S2, S3 obtained (Scheme 1). The first test was the water solubility test which is considered as the first indication of hydrolyzing the water insoluble chitosan to its water soluble chito-oligomers. Such data can give clear cut evidence of the hydrolysis of chitosan under the abovementioned reaction conditions. The second test performed is the light absorbance of the solution that results from the reaction of soluble obtained sample (S1, S2, and S3) with ninhydrin; the high absorbance is an indication to the high chito-oligomers content. Comparing the light absorbance data obtained from S1, S2, and S3 with the light absorbance of a chitosan (starting material) and glucosamine (pure monomer) samples gave an evidence of the presence or absence of chito-oligomers in the tested samples. The third test was the % loss upon ignition which is an indication of the % organic matter in the different samples collected within the work-up of the chitosan hydrolysis reaction (S1, S2, S3) in order to distinguish it from the zeolite and salt contents of the samples obtained from the hydrolysis reaction.

Surprisingly, very little content of the chito-oligomers was observed in the product sample S1 (Scheme 1) which is obtained from evaporation of the solution that results after centrifuging the reaction mixture. For example, 1 (S1) was found to be water insoluble, has a low absorbance in the ninhydrin test (0.014), and has a low % loss upon ignition (30%) (Table 3).

The solid product of the centrifugation of the reaction mixture was then neutralized to pH = 8 using 10% NaOH solution. After shaking, centrifugation, and evaporation of clear solution, solid product S2 (Scheme 1) was obtained. The product 1 (S2) was found to be water-soluble, has low absorbance in the ninhydrin test (0.004), and has a low % loss upon ignition (13%) (Table 3). These results indicated that there is very little content of chito-oligomers in this product and most of the solid is expected to be NaCl salt from the neutralization reaction.

As a third trial to find the chito-oligomers, the solid product of the centrifugation of the reaction mixture was strongly stirred (cortex) with distilled water (instead of 10% NaOH solution, as in the above paragraph), followed by centrifugation to remove the adsorbent material. Evaporating the obtained solution gave a light beige solid S3 (Scheme 1). S3 was found to be water- soluble, showed high absorbance in the ninhydrin test (absorbance = 2.4 using 3 Ǻ zeolite, 2.9 using 5Ǻ zeolite, and 2.3 using 10Ǻ zeolite) compared with chitosan with absorbance = 0.003, and it has also high % loss upon ignition (81% using 3Ǻ zeolite, 68% using 5Ǻ zeolite, 67% using 10Ǻ zeolite) as an indication of organic compound content (Table 3).

Actually, the three types of zeolites employed in the present work were used in order to investigate the effect of pore dimension of zeolite on the hydrolysis reactions and products. The detailed properties of zeolites are given in Table 2. However, the type of zeolite did not affect chito-oligomers obtained significantly as indicated by the results in Table 3 and as indicated also by their mass spectra. This may be due to that chito-oligomers are adsorbed on the surface rather than in the pores of zeolites.

As shown in Table 2, HZSM-5 (5 Å) is distinguished by a high SiO_2_/Al_2_O_3_ molar ratio, so it is a hydrophobic zeolite [43]. It was reported to be stable in acidic solution (1 M HCl) [43,44] and stable in aqueous hot water (150°–500°, 5–17 bar) [45] relative to other kinds of zeolites. In our experiments, it was observed that molecular sieve zeolites (3 and 10 Å) partially decomposed into solution as reflected by their orange color while HZSM-5 zeolite (5 Å) remained stable.

The preliminary results of study of S3 product using water solubility, the ninhydrin test, and the % loss upon ignition indicated that after centrifugation of the acidic hydrolysis reaction mixture; chitosan was hydrolyzed to chito-oligomers which were completely adsorbed on zeolite. The adsorbed chito-oligomers could be retrieved by sonicating the solid product in deionized water. Thus, the hydrolyzed product S3, which contains chito-oligomers and decomposed zeolites, was chosen for further characterization and further study in the following sections.

### 2.2 Characterization of Chito-Oligomers

#### 2.2.1. DSC Study

**Figure 2:**
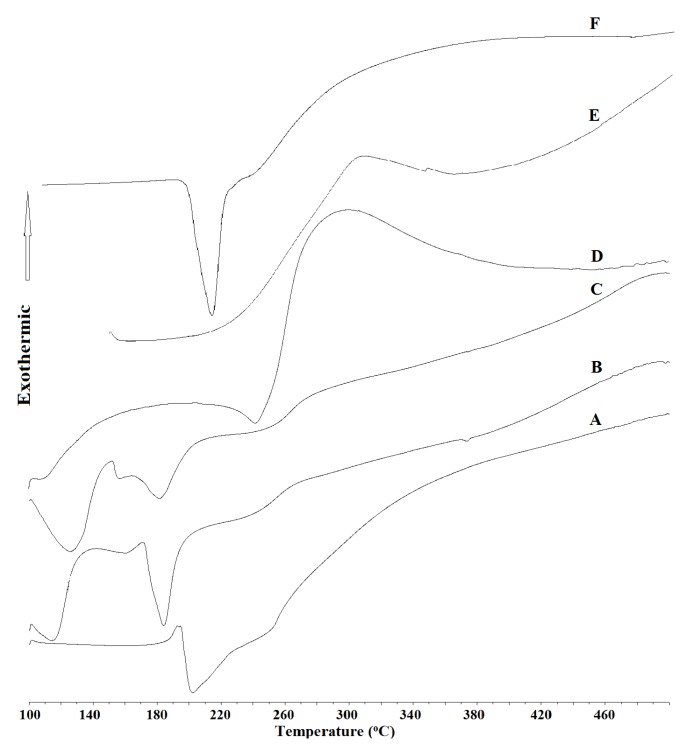
DSC of the hydrolyzed products (**A**) 1 (S3/5 Å-Zeolite); (**B**) 2 (S3/10 Å-Zeolite); (**C**) 3 (S3/3 Å-Zeolite) and (**D**) 4 (S3/No zeolite); (**E**) chitosan; and (**F**) glucosamine.

The DSC diagrams of chitosan and glucosamine are given in Figure 2. Chitosan has an exothermic peak at 350 °C while glucosamine has a sharp endothermic peak at 210 °C, followed by decomposition. The hydrolyzed product 1 (S5/5 Å-Zeolite) was found to have a broad endothermic peak at a temperature close to that of glucosamine (Figure 2), followed by decomposition. A similar behavior was observed in the case of hydrolyzed products 2 (S3/10 Å-Zeolite) and 3 (S3/3 Å-Zeolite), but an additional endothermic peak at 120 °C, corresponding to the onset of the evaporation of bound water, was observed as well (Figure 2) [46].

#### 2.2.2. Mass Spectra

**Figure 3:**
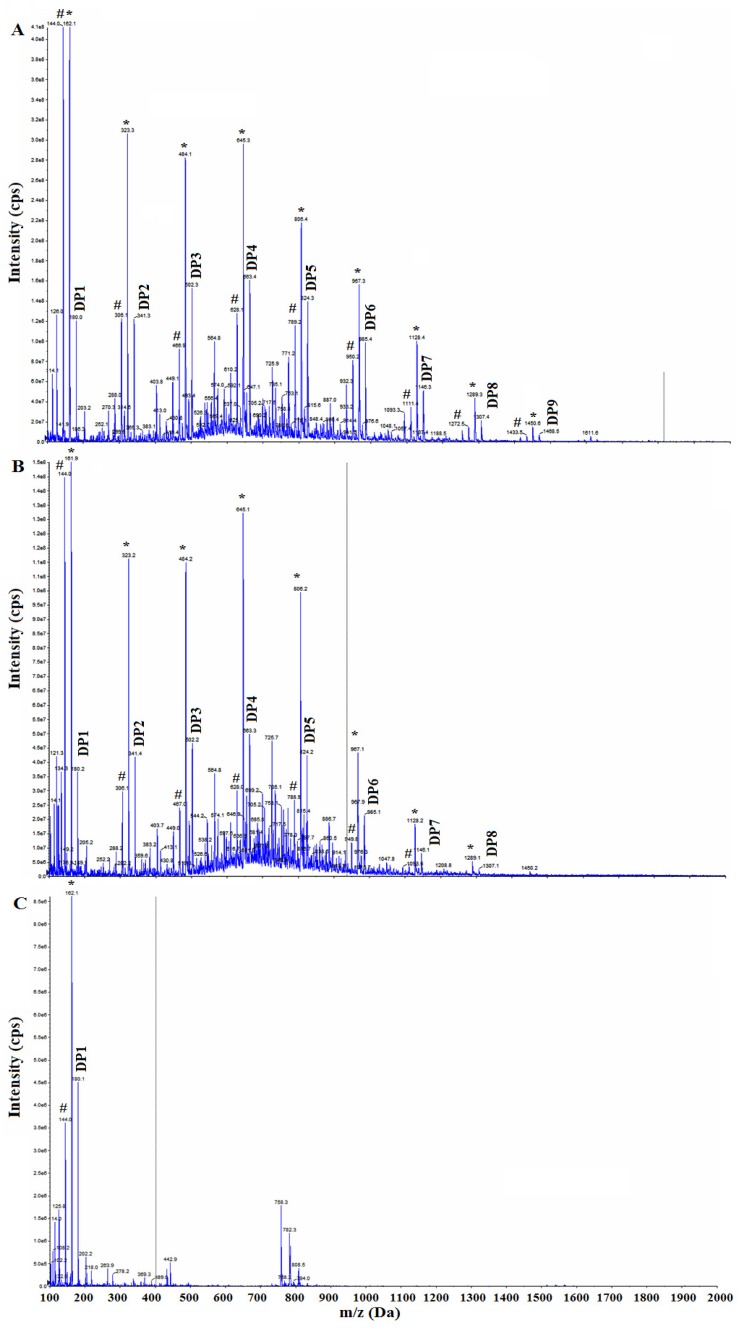
Mass spectra of hydrolyzed products (**A**) 1 (S3/5 Å-Zeolite); (**B**) 4 (S3/No zeolite); and (**C**) glucosamine.

The theoretical molar mass values of chito-oligomers are: DP1 = 179.16, DP2 = 340.31, DP3 = 501.45, DP4 = 662.60, DP5 = 823.74, DP6 = 984.89, DP7 = 1146.03, DP8 = 1307.18, DP9 = 1468.32. The masses obtained experimentally are shown in Figure 3 for the products 4 (S3/No zeolite) and 1 (S3/5 Å-Zeolite). Similar values were obtained for all S3 products which gave positive tests in Table 2. Note that there are peaks resulting from the dehydration of the chito-oligomers (marked by * and # in Figure 3) and are due to the loss of one and two water molecules. The dehydration peaks were a result of the analysis method because these peaks were also observed in the case of the glucosamine standard (Figure 3). Interestingly, the intensity of the *m/z* peaks of the chito-oligomers decreases in the order of increasing DP. The highest DP chio-oligomer detected was that of DP = 9.

#### 2.2.3 Recrystallization of Chito-Oligomers

The aim of recrystallization was to remove the high molar mass chitosan from the chito-oligomers. The former are insoluble in neutral or basic medium while the latter are soluble. Thus, addition of base is necessary to precipitate the high molar mass chitosan. Ammonia was selected because the formed ammonium chloride replaces the chito-oligomers ammonium salts and desorbs them from chitosan and zeolite surfaces. The properties of recrystallized products are given in Table 4.

The low % yield of chito-oligomers in the absence of zeolite suggests that zeolites function as adsorbents for the produced chito-oligomers and causing shift of the hydrolysis equilibrium reaction forward and, thus, increasing the yield of the hydrolyzed chito-oligomers.

It is worth to mention that the % yields obtained cannot be compared with those in the literature because neither our study nor previous studies give cut evidences for the purity of chito-oligomers products.

## 3. Experimental Section

### 3.1. Materials

Chitosan (molecular weight ~250 kDa and DDA ~93%) was obtained from Homg Ju, HZSM-5 zeolite (5 Å) and molecular sieves (10 Å and 3 Å) were obtained from Across Organics and Merck, respectively. HCl (37%) was from Merck and sodium hydroxide was from GCC. The ammonia solution (25%) was obtained from Scharlue. Ethanol (Absolute) was purchased from GCC.

### 3.2. Methods

#### 3.2.1. Hydrolysis of Chitosan

A 2.0 g sample of chitosan was mixed with 85 mL deionized water using a magnetic stirrer. A 4.0 g of zeolite (HZSM-5, molecular sieves 10 Å and 3 Å) was then added followed by the addition of 15 mL of concentrated HCl solution to give a final concentration of 1.8 M HCl solution. The mixture was then heated under reflux at 100 °C with stirring for 2 h. The mixture was then cooled to room temperature and divided into two portions and centrifuged (Hermle Z 320) at 4500 rpm for 15 min. The two solutions in the 50 mL centrifuge tubes were combined and evaporated for dryness (S1, Scheme 1) using a rotary evaporator. Distilled water was then added to the solid obtained in the two centrifuge tubes and the tubes were shaken using vortex (Labinco). The solution in one centrifuge tube was neutralized to pH = 8 using 10% NaOH solution. The resultant two mixtures were centrifuged at 4500 rpm for 15 min. The two resultant solutions were separated and evaporated in a rotary evaporator. The solids obtained from base and acid retrieval were designated S2 and S3, respectively (Scheme 1).

#### 3.2.2. The Ninhydrin Test

A 0.10 g sample of the solids obtained from the hydrolysis procedure (2.2.1) was dissolved in 20 mL deionized water. A 2.0 mL portion was mixed with a buffer of KH_2_PO_4_ (0.2 M, pH 6) and 2.0 mL 0.8% ninhydrin. The solution was then heated to 80 °C in a water bath for 1 h. The absorbance of the resultant solution was measured using a UV-VIS Spectrophotometer (PG Instruments Ltd. T80).

#### 3.2.3. The % Loss upon Ignition Measurement

A 0.1 g sample measured to the nearest of 0.0001 g was ignited in a crucible using a Bunsen burner and the total mass of the crucible and the residue was then measured. The empty crucible was previously ignited and its mass measured.

#### 3.2.4. DSC Measurements

DSC measurements of the samples; in the range of 100–500 °C; were carried out in aluminum crucibles using Mettler Toledi DSC-1 Star System. The rate of heating was 10 °C/min.

#### 3.2.5. Mass Spectra

The mass spectra of the samples were measured using AB Sciex LC-MSMS API-3200 LC-MS instrument. A 0.1 g sample was dissolved in 10.0 mL deionized water and introduced into the LC-MS instrument with a rate of 10 µL/min. The ionization source was turbo spray; the ion spray voltage was 5500. Typically, very little fragmentation or chemical reactions occur in this kind of soft ionization technique. The mass spectra were recorded in the range from 100 to 2000 *m/z* using MCA positive polarity mode where 60 scans were collected in 2.0 min. The step size was 0.1 Da. The peak area was obtained for all the peaks directly from the instrument.

#### 3.2.6. Recrystallization Using Ammonia

A 1.0 g sample of the crude solid (S3, Scheme 1) obtained in procedure 2.2.1 was dissolved in 50 mL deionized water followed by the addition of 1.5 mL of 25% NH_3_. The mixture was then centrifuged at 4500 rpm for 15 min, the solid precipitated was excluded and the solution was evaporated in the rotary evaporator.

#### 3.2.7. Recrystallization Using Ethanol and Ammonia

A 1.0 g sample of the crude solid (S3, Scheme 1) obtained in procedure 2.2.1 was dissolved in 25 mL deionized water. Then 25 mL absolute ethanol was added followed by the addition of 0.5 mL of 25% NH_3_. The mixture was then centrifuged at 4500 rpm for 15 min, the solid precipitated was excluded and the solution was evaporated in the rotary evaporator.

## 4. Conclusions

The method developed in the present study offers a simple process for preparation and separation of chito-oligomers from chitosan. The zeolites function as adsorbents for the produced chito-oligomers and cause a shift of the hydrolysis equilibrium reaction forward and, thus, increase the yield of the hydrolyzed products. More research is required on the hydrolysis of chitin/chitosan to its corresponding chito-oligomers using adsorbents other than zeolites. Studying the kinetics of the hydrolysis reaction in the presence of zeolites and the biological evaluation of the obtained chito-oligomers is also necessary to inspect the potential of these chito-oligomers.

## Figures and Tables

**Figure 4:**
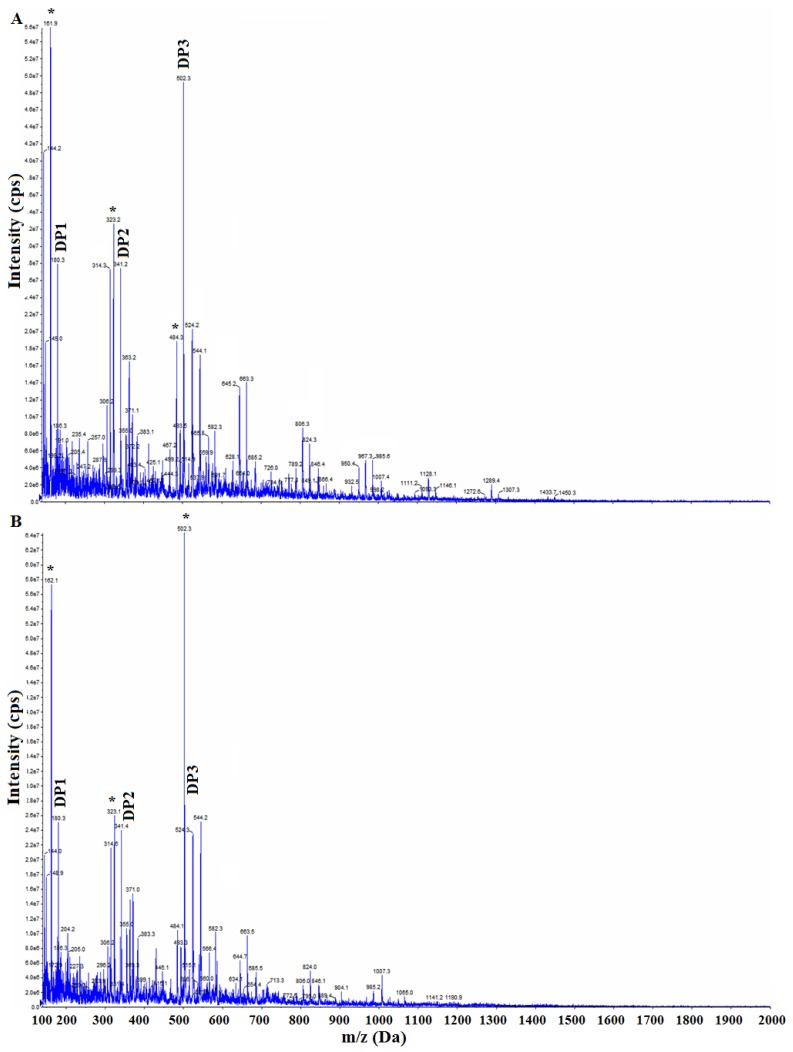
Mass spectra of recrystallized hydrolyzed product 4 (S3/No zeolite) using (**A**) ammonia-ethanol and (**B**) using ammonia alone.

**Table 1:** Summary of reagent, reaction conditions, and final product characterization of a number of described hydrolysis reactions of chitosan using concentrated HCl (12 M).

DA of Starting Chitosan	Reaction Conditions	Final Product	Characterization	Reference
DA ~0%	72 °C, 1.5 h	DP = 2–12	Mass spectroscopy	[16]
DA < 10%	70 °C, 4 h	DP = 6–16	HPLC-light scattering detector	[19]
DA = 12%	72 °C, 0.5 h	DP = 3–16	Mass spectroscopy	[22]
DA ~0%	72 °C, 0.5–3 h	DP < 40	Size exclusion chromatography-refractive index detector	[23]

DA: Degree of acetylation; DP: Degree of polymerization.

**Table 2:** Properties of zeolites used as adsorbents in the present work.

Property	HZSM-5 *	Molecular Sieves ** Beads 0.3 nm	Molecular Sieves ** Beads 1.0 nm
Pore diameter (Å)	5	3	10
Composition	Aluminosilicate	Potassium sodium aluminum silicate	Sodium aluminum silicate
	Na < 700 ppm		
Main application	Petroleum industry-hydrocarbon isomerization	Water adsorption	Chloroform, carbon tetrachloride, benzene adsorption
SiO_2_/Al_2_O_3_ molar ratio	400–570	2	3
	50 [39]		
	76 [40]		
Total acid density	0.2–0.26 [40]	-	-
	0.11–0.64 [41]		
BET total surface area (m^2^/g)	300	800	
	364 [42]		
	389 [39]		
	392 [40]		
	826–1142 [41]		
Micropore surface area (m^2^/g)	303 [39]	-	-
	359 [40]		
	728–1036 [41]		
Mesopore surface area (m^2^/g)	85 [39]	-	-
	33 [40]		
	98–106 [41]		
Total pore volume (cm^3^/g)	0.22 [40]	0.30	
	0.19–0.25 [41]		
Micropore volume (cm^3^/g)	0.09 [42]	-	-
	0.12 [39]		
	0.16 [40]		
	0.10–0.14 [41]		
Mesopore volume (cm^3^/g)	0.12 [42]	-	-
	0.04 [39]		
	0.06 [40]		
	0.09–0.1 [41]		

*: Values given without references were obtained from Acros Organics specifications; **: Values given without references were obtained from Merck specifications.

**Table 3:** Properties of glucosamine, chitosan, and the solids obtained from the hydrolysis of chitosan (S1, S2, and S3) using diluted HCl in the presence of zeolites.

Sample	Product Number	Solubility	Absorbance of Ninhydrin Test	% Loss Upon Ignition	DSC Peaks (°C)
Glucosamine		+	2.6	68	210–220
Chitosan		−	0.003	75	350
HCl(5Å-Z)-Acid retrieval	1 (S3)	+	2.9	68	200–230
					230–250
HCl(5Å-Z)-Base retrieval	1 (S2)	+	0.004	13	No peaks were detected
HCl(5Å-Z)-First filtrate	1 (S1)	−	0.014	30	Not determined
HCl(10Å-Z)-Acid retrieval	2 (S3)	+	2.3	67	110–120
					180–190
					230–250
HCl(10Å-Z)-Base retrieval	2 (S2)	+	0.011	21	No peaks were detected
HCl(3Å-Z)-Acid retrieval	3 (S3)	+	2.4	81	110–140
					150–160
					180
					220–250
HCl(3Å-Z)-Base retrieval	3 (S2)	+	0.006	10	No peaks were detected
HCl-Acid retrieval	4 (S3)	+			240–250

**Table 4:** Properties of recrystallized chito-oligomers.

Sample	Recrystallization Reagents	Solubility	% Yield	DSC Peaks (°C)
1 (S3/5 Å-Zeolite)	NH_3_	+	47	
1 (S3/5 Å-Zeolite)	Ethanol-NH_3_	+	38	220, 240, 280
2 (S3/10 Å-Zeolite)	NH_3_	+	80	200–210 (NH_4_Cl)
2 (S3/10 Å-Zeolite)	Ethanol-NH_3_	+	96	220
3 (S3/3 Å-Zeolite)	NH_3_	+	68	180–200 (NH_4_Cl)
3 (S3/3 Å-Zeolite)	Ethanol-NH_3_	+	56	220
4 (S3 No zeolite)	NH_3_	+	12	NH_4_Cl
4 (S3 No zeolite)	Ethanol-NH_3_	+	6.0	NH_4_Cl

The % yield of products (based on starting 2.0 g chitosan) after recrystallization in the absence of zeolites for the hydrolyzed product 4 (S3/No zeolite) were much lower than those in the presence of zeolites. Furthermore, The DSC diagrams of these samples showed only those peaks of ammonium chloride (200 °C, 340–360 °C broad). However, the mass spectra of these samples were very interesting (Figure 4) because chito-oligomers with DP from 1 to 3 were obtained as major products.
